# Spontaneous Uterine Rupture Secondary to Pyometra in a Postmenopausal Patient

**DOI:** 10.7759/cureus.82398

**Published:** 2025-04-16

**Authors:** Oscar Antonio Regalado Morales, José Luis Herrera Alanís, Luis Alberto Solís García, Abraham Alejandro Zorrilla Silva, Marcelo Valdés Hernández

**Affiliations:** 1 Radiology, Hospital Regional Monterrey, Instituto de Seguridad y Servicios Sociales de los Trabajadores del Estado, Monterrey, MEX

**Keywords:** abdominal radiology, acute abdomen, pelvic computed tomography, pyometra, spontaneous uterine rupture

## Abstract

Pyometra is the accumulation of purulent material in the endometrial cavity. It is an uncommon condition in postmenopausal women that can lead to uterine rupture, a complication with high mortality if not treated promptly. Imaging studies, particularly computed tomography (CT), play a fundamental role in diagnosing this condition and excluding more common pathologies. We present the case of an 86-year-old female patient with an acute abdomen secondary to uterine rupture due to pyometra diagnosed via CT.

## Introduction

Acute abdomen is one of the leading causes of hospital admission in elderly individuals, accounting for up to 25% of emergency visits [1]. Among its primary etiologies in the geriatric population, biliary tract diseases are the most frequent, followed by nonspecific abdominal pain, malignant neoplasms, intestinal obstruction, and complicated hernias [2]. However, due to the nonspecific nature of clinical findings, up to 40% of patients receive an incorrect diagnosis, delaying treatment and increasing mortality [3].

Gynecological causes are not among the most common etiologies of acute abdomen in this age group; therefore, when they occur, their diagnosis presents a challenge that may lead to adverse health outcomes for patients [2].

In this study, we present the case of a postmenopausal patient with an acute abdomen secondary to uterine rupture due to pyometra.

## Case presentation

An 86-year-old female patient presented to the emergency department with diffuse abdominal pain, rated 8/10 on the visual analog scale (VAS). Physical examination revealed tenderness on palpation and signs of peritoneal irritation. Laboratory studies showed leukocytosis with neutrophilic predominance (Table 1).

**Table 1:** Initial laboratory tests.

Laboratory Test	Result	Unit	Reference
Leukocytes	28.1	10^3/uL	4.5-10.5
Neutrophils	26.9	10^3/uL	2.10-6.10
Hemoglobin	12.1	g/dL	12-16
Hematocrit	37	%	37-47
Platelets	368	10^3/uL	140-400
Glucose	89	mg/dL	74-106
Creatinina	1.2	mg/dL	0.50-1.30

**Figure 1:**
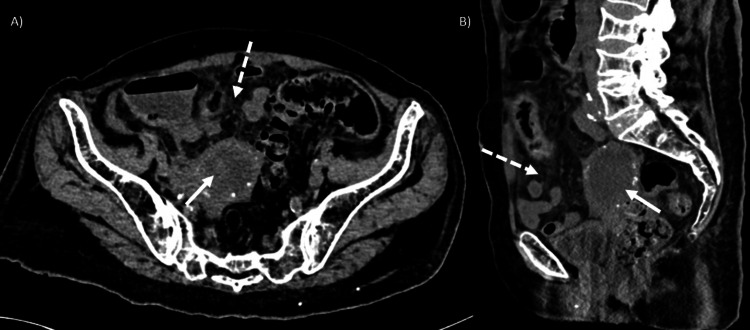
Computerized tomography in a simple phase of the abdomen. Axial (A) and sagittal (B) CT scans show a heterogeneous-appearing uterus with hypodense material in the endometrial cavity (thick arrow), associated with increased adjacent fat density (thick arrow).

Due to the clinical presentation, an initial diagnosis of acute diverticulitis was considered. A non-contrast abdominal CT scan was performed, revealing a heterogeneous-appearing uterus with hypodense material in the endometrial cavity, associated with increased adjacent fat density (Figure 1). No signs of diverticulitis or intestinal perforation were identified.

**Figure 2:**
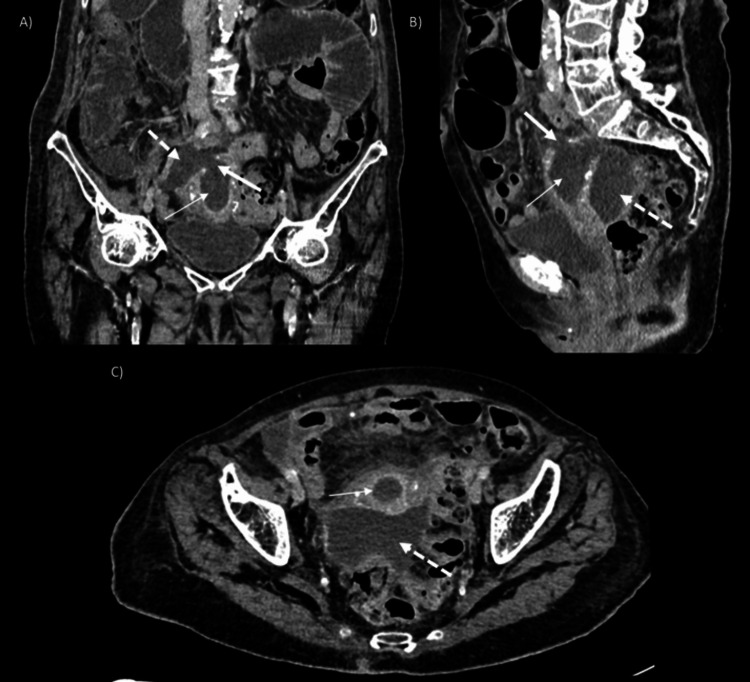
Computerized tomography in the venous phase of the abdomen. Coronal (A), sagittal (B) and axial (C) CT scans show distension of the endometrial cavity (thin arrow) with a 1.5-cm diameter defect in the uterine wall at the fundus (thick arrow) communicating with an abscess in the pouch of Douglas (dotted arrow).

A conservative management approach was initially chosen; however, in the following days, the patient's abdominal pain worsened, prompting a new contrast-enhanced CT scan. This time, the scan showed distension of the endometrial cavity with a defect in the uterine wall at the fundus, communicating with an abscess in the pouch of Douglas, findings consistent with uterine perforation secondary to pyometra (Figure 2).

**Figure 3:**
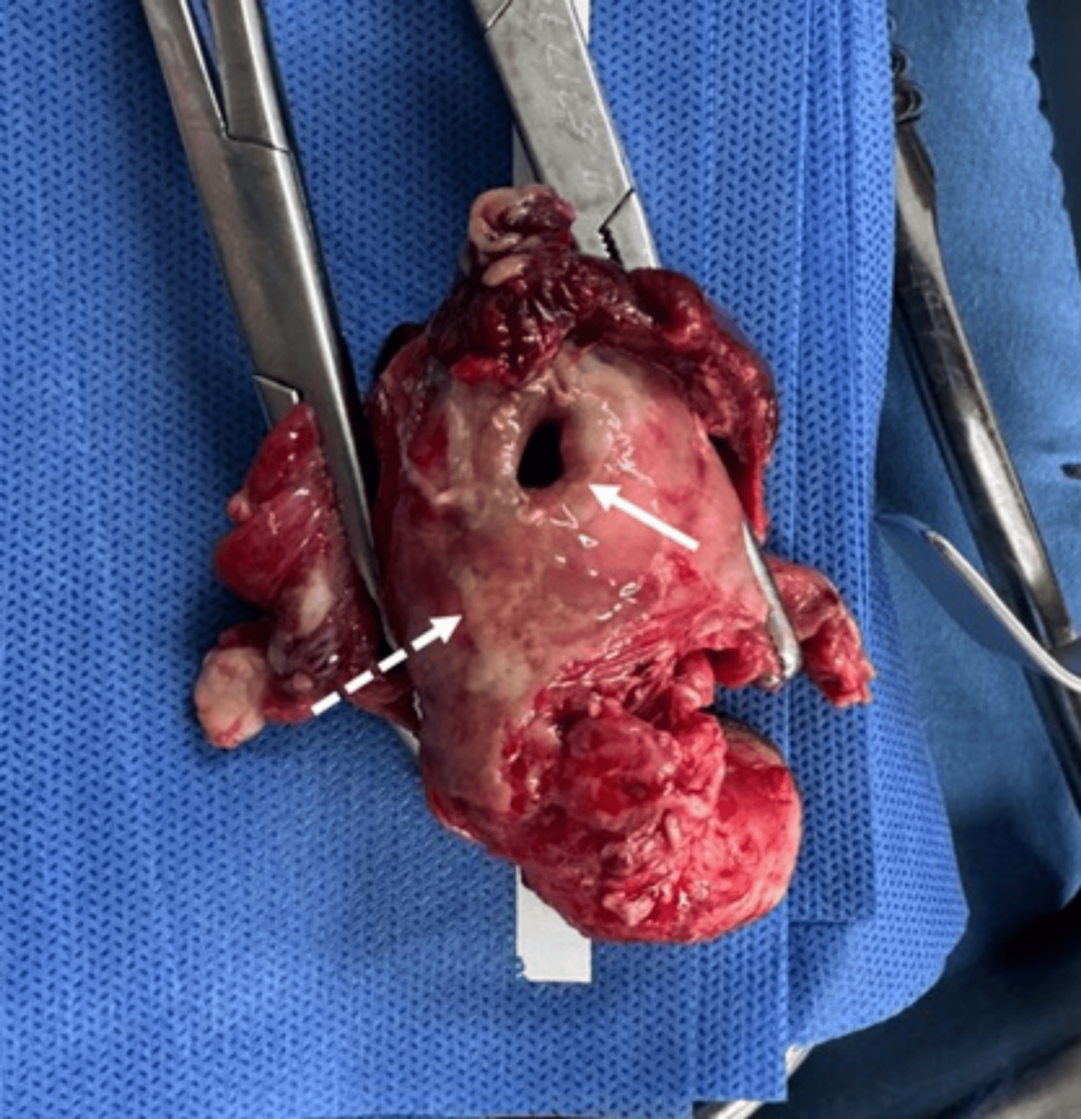
Gross photograph of the uterus after total hysterectomy with bilateral salpingo-oophorectomy. This image shows a defect in the uterine wall at the fundus, consistent with rupture (thick arrow). Additionally, purulent exudate is seen covering the uterine serosa (dotted arrow).

Given these findings, an emergency laparotomy was performed, confirming a perforation at the uterine fundus associated with an abscess in the pelvic cavity. A total hysterectomy with bilateral salpingo-oophorectomy and abscess drainage was performed, with a total volume of 200 cc (Figure 3).

## Discussion

Uterine perforation or rupture refers to a defect in the myometrium that creates communication between the endometrial and abdominal cavities. While both terms can be used interchangeably, "rupture" is typically preferred when the cause is non-iatrogenic. The etiology is classified into gravid and non-gravid causes, with pyometra being one of the rarest non-gravid causes. This condition is characterized by the accumulation of purulent material within the endometrial cavity [4]. Uterine rupture secondary to pyometra is an extremely rare complication, with an estimated incidence of 0.01% to 0.05% [5].

This condition primarily affects elderly women, with a mean age of 75 years [6]. It results from obstruction of the uterine drainage pathway, which may be secondary to benign or malignant pathologies, including cervical stenosis, post-radiation changes, pelvic inflammatory disease, polyps, and leiomyomas [7]. The obstruction leads to endometrial cavity distension, ultimately causing necrosis of the uterine wall and subsequent perforation, with the uterine fundus being the most affected site due to its lower vascularity [4]. The main isolated microorganisms are *Escherichia coli*, *Staphylococcus aureus*, and *Streptococcus* species [8].

Clinically, pyometra is associated with a classic triad of purulent vaginal discharge, postmenopausal bleeding, and abdominal pain. However, up to 50% of cases may be asymptomatic [9]. When uterine rupture occurs, patients present with signs of an acute abdomen and peritoneal irritation [4].

The preoperative diagnosis of uterine rupture is complex, often requiring emergency laparotomy for confirmation due to the nonspecific nature of clinical findings [8]. Imaging studies play a fundamental role in evaluating this pathology, with ultrasound and computed tomography being the most commonly used modalities. On ultrasound, pyometra appears as a heterogeneous collection within the endometrial cavity, occasionally containing echogenic foci in the non-dependent portion of the uterus with posterior dirty shadowing, indicative of gas. On CT, findings are similar, with distension of the endometrial cavity and possible presence of intrauterine gas. Uterine rupture is identified as a defect in the uterine wall, frequently associated with abscess formation in the abdominal cavity and, in some cases, the presence of free air [4].

The treatment of pyometra involves drainage of the endometrial cavity along with antibiotic therapy. However, if uterine rupture is suspected, emergency laparotomy is essential, as this condition carries a mortality rate of up to 31% [10].

## Conclusions

Uterine rupture secondary to pyometra is a rare cause of abdominal pain in elderly patients. Its clinical manifestations and physical examination findings are often nonspecific, leading to potential misdiagnosis with more common pathologies and delayed treatment. Imaging studies, particularly abdominal computed tomography, play a key role in diagnosis, allowing not only the identification of this entity but also the exclusion of other possible causes. Characteristic findings include discontinuity of the endometrial wall associated with abscess formation, guiding prompt diagnosis and appropriate management.

## References

[1] Dadeh AA, Uppakarnnuntakul W (2024). Factors associated with serious abdominal conditions in geriatric patients visiting the emergency department. BMC Emerg Med.

[2] Martinez JP, Mattu A (2006). Abdominal pain in the elderly. Emerg Med Clin North Am.

[3] Nagaratnam N, Nagaratnam K, Cheuk G (2017). Acute abdominal pain in the elderly. Geriatric Diseases.

[4] Aboughalia H, Basavalingu D, Revzin MV, Sienas LE, Katz DS, Moshiri M (2021). Imaging evaluation of uterine perforation and rupture. Abdom Radiol (NY).

[5] Mahmood U, Aziz MU, Munawar M, Amjad L, Azmat CE, Siddique S (2025). Uterine rupture due to pyometra and imperforate hymen in a 7-year-old girl: a case report. J Pediatr Surg Case Rep.

[6] Kitai T, Okuno K, Ugaki H, Komoto Y, Fujimi S, Takemura M (2014). Spontaneous uterine perforation of pyometra presenting as acute abdomen. Case Rep Obstet Gynecol.

[7] Mohd Hanapiah F, Ismail ZK, Puteh O, Aziz ME (2024). Computed tomography findings in a case of uterine rupture as a complication of pyometra. Cureus.

[8] Kishe A, William D, Moshi PG, Marandu AA, Mremi A, Kimario AA (2025). Spontaneous uterine perforation due to pyometra: A rare cause of acute abdomen in a postmenopausal woman. Int J Surg Case Rep.

[9] Díaz de la Noval B, Gómez Alarcón A, González de Merlo G (2017). Presentación atípica de la piómetra en la mujer anciana [article in Spanish]. Clin Invest Ginecol Obstet.

[10] Franklin NM, LaFree A, Gocke S, Corbett B, Wituck P, Nene R (2024). Acute pyometra in an elderly female patient: A case report. JEM Rep.

